# Cytogenetic and Molecular Characteristics in Adult Hispanic Acute Myeloid Leukemia Patients From Puerto Rico

**DOI:** 10.7759/cureus.70388

**Published:** 2024-09-28

**Authors:** Daniel H Jiang, Hongyu Ni, Mario Curti, Vu Phan, Jie-Gen Jiang, Lihong Wu

**Affiliations:** 1 Technology, Troy Technology High School, Fullerton, USA; 2 Pathology and Laboratory Medicine, Cedars-Sinai Medical Center, Los Angeles, USA; 3 Hematology and Oncology, University of California, Irvine (UCI) Health, Los Alamitos, USA; 4 Pathology, University of California, Irvine (UCI) Health, Los Alamitos, USA; 5 Hematology and Oncology, City of Hope, Long Beach, USA

**Keywords:** acute myeloid leukemia, cytogenetic abnormality, hispanic population, molecular mutation, puerto rico

## Abstract

The cytogenetic and molecular heterogeneity of acute myeloid leukemia (AML) is characterized as a contributing factor in the disparity of treatment outcomes and clinical outcomes seen among ethnic and racial groups. In this study, we have retrospectively evaluated the karyotypes of 800 adult Hispanic AML patients from Puerto Rico (PR). Acute promyelocytic leukemia with *PML-RARA* is the most common recurrent cytogenetic abnormality, compatible with previously published results. Among these AML patients, 163 patients had 21 gene panels performed. Twenty-six (15.95%) patients showed no detectable mutations, and 137 patients (84.05%) showed at least one mutation. Compared with previously published data from other examined Hispanic AML populations in the United States, mutational frequencies of these 21 genes, except for *ASXL1*, *WT1*, and *KRAS*, show no significant difference. This is the largest study to date about the landscape of cytogenetic and molecular abnormalities in Hispanic AML patients and a first report regarding the frequencies of these abnormalities in Puerto Rican Hispanic AML patients.

## Introduction

Acute myeloid leukemia (AML) is a hematopoietic stem cell malignancy with uncontrolled clonal expansion of myeloid progenitor cells. The clinical outcome of AML patients is associated with many known risk factors, including age, race, clinical characteristics, sociodemographic status, disease pathobiology, and comorbidities, as well as cytogenetic abnormalities and molecular mutations. At diagnosis, most patients with AML harbor at least one chromosome aberration in their marrow blasts, and more than 95% of AML cases are confirmed to have at least one somatic mutation, with approximately a dozen molecular mutations identified per AML sample, including an average of three driver mutations [1].

Racial differences in clinical characteristics and outcomes have been identified in cancer patients. The clinical and biological heterogeneity of AML is a characteristic that is currently under investigation as a contributing factor in the disparity of treatment outcomes and clinical outcomes seen among different ethnic and racial groups [2]. There is a relative dearth of data evaluating AML across different ethnic and racial groups [3], despite AML being found to be more common in Hispanics than in non-Hispanic whites [4] and Hispanics being the second largest ethnic group in the United States. There were also no differences in incidence, regardless of whether the patients were native or foreign-born.

The few published articles available have highlighted a number of cytogenetic and molecular differences in AML between Hispanics and non-Hispanic whites. Dour et al. [5] revealed that Hispanics with AML have a higher likelihood of the *APL* subtype. Monge et al. [3] found increased *MLL PTD* gene mutations, a poor-risk abnormality, in Hispanic patients and increased *IDH1* mutations in non-Hispanic whites but found no significant differences. In a follow-up study, Bradley et al. [6] found that *IDH1* mutations are more common in non-Hispanic whites, and *WT1* mutations are more common in Hispanics. Darbinyan et al. [2] revealed significantly higher mutational frequencies of *ASXL1* and *TET2* genes in Hispanics than in non-Hispanic whites.

In the largest study, we have retrospectively analyzed adult Hispanic AML patients from Puerto Rico (PR) and identified 800 patients with cytogenetic tests and 163 patients with 21 molecular markers performed. The aim of our current study was to evaluate the prevalence of prognostic cytogenetic and molecular markers in Hispanic AML patients from PR.

## Materials and methods

Patients

We conducted a retrospective investigation on patients diagnosed with AML in Genoptix Medical Laboratory, Carlsbad, California, USA (now NeoGenomics Laboratories) between 2008 and 2018. This retrospective study was approved by Sterling IRB (ID: 6173). We identified 800 adult patients from PR who had AML. The diagnosis of AML was established according to the criteria proposed by the World Health Organization (WHO).

Cytogenetic analysis

Conventional cytogenetic analysis was performed on G-banded metaphase cells prepared from unstimulated 24-hour and 48-hour bone marrow aspirates cultured using standard techniques. At least 20 metaphases with good-quality banding were evaluated for each case when satisfactory cell cultures were available. A clonal cytogenetic abnormality is defined as the same numerical gain or structural abnormalities in at least two metaphases or the same numerical loss in at least three. A complex abnormal karyotype is defined as three or more cytogenetic abnormalities. The karyotype was documented according to the International System for Human Cytogenetic Nomenclatures (ISCN 2013).

Molecular profile by next-generation sequencing

The next-generation sequencing (NGS) profile, the AML molecular profile, was used to detect key gene mutations in AML. Genomic DNA was isolated from bone marrow aspirate. DNA sequence of targeted regions of the *ASXL1*, *BCOR*, *CEBPA*, *DNMT3A*, *EZH2*, *IDH1*, *IDH2*, *KIT*, *KRAS*, *NPM1*, *NRAS*, *PHF6*, *RUNX1*, *SF3B1*, *SRSF2*, *STAG2*, *TET2*, *TP53*, *U2AF1*, *WT1*, and *ZRSR2* genes was determined using NGS technology. The molecular alterations within each gene were analyzed through proprietary bioinformatic software and interpreted in conjunction with reference databases such as COSMIC, ClinVar, gnomAD, and dbSNP. Quality control metrics included a minimum input of 20 ng, with an optimal input of 100 ng of genomic DNA and an average mean sequencing depth of 500× coverage. The limits of detection (LOD) were 5% for SNV, 10% for Indels, ≥6 copies for gene amplifications, and ≤0.3 copies for homozygous gene deletions. Insertions greater than 15 nucleotides and deletions greater than 52 nucleotides may not be detected. Benign sequence variants were not reported.

Mutations in *FLT3* and *MLL*

Mutations in *FLT3* and *MLL* were not included in the above molecular profile panels and were performed separately. Nucleic acid was isolated from bone marrow aspirate. Internal tandem duplication (*FLT3-ITD*) and exon 20 tyrosine kinase domain mutations (*FLT3-TKD*) in the *FLT3* gene were analyzed by using fragment-length analysis and PCR. Positive results identify the presence of *TKD* mutations or report ITD results quantitatively as an allelic ratio. Partial tandem duplication of *MLL* (*MLL-PTD*) was screened by reverse-transcriptase PCR and confirmed by real-time quantitative PCR.

Statistical analysis

Fisher’s exact test was used to analyze differences in the distribution of molecular abnormalities among different AML patient populations. Multiple comparison corrections were performed to control the false discovery rate (FDR) using the Benjamini-Hochberg procedure, and then significance was determined accordingly.

## Results

Cytogenetic results

We identified 800 Hispanic patients (48% males, 52% females) diagnosed with AML from PR and analyzed them for cytogenetic profiling. Median age was 63 years (range: 50-76). The female median age was 61 years (range: 53-74), and the male median age was 65 years (range: 50-76). The patients’ recurrent cytogenetic abnormalities were summarized in Table 1.

**Table 1:** Recurrent cytogenetic abnormalities in Hispanic acute myeloid leukemia patients from Puerto Rico Total patients = 800, males = 386, females = 414

Recurrent cytogenetic abnormality	Male, number (%)	Female, number (%)	Total number (%)
t(15;17)(q24;q21) *PML/RARA*	41 (10.62%)	51 (12.23%)	92 (11.5%)
inv(16)(p13.1q22) *CBFB/MYH11*	15 (3.88%)	19 (4.56%)	34 (4.25%)
t(8;21)(q22;q22) *RUNX1/RUNX1T1*	10 (2.59%)	17 (4.08%)	27 (3.38%)
t(9;22)(q34;q11)* BCR/ABL1*	3 (0.78%)	4 (0.97%)	7 (0.88%)

The most frequent recurrent cytogenetic abnormalities include t(15;17)(q24;q21) *PML/RARA* (11.5% in total: 10.62% in males and 12.23% in females), inv(16)(p13.1q22) *CBFB/MYH11* (4.25% in total: 3.88% in males and 4.56% in females), t(8;21)(q22;q22) *RUNX1/RUNX1T1* (3.38% in total: 2.59% in males and 4.08% in females), and t(9;22)(q34;q11) *BCR/ABL1* (0.88% in total: 0.78% in males and 0.97% in females).

Molecular mutation results

**Figure 1:**
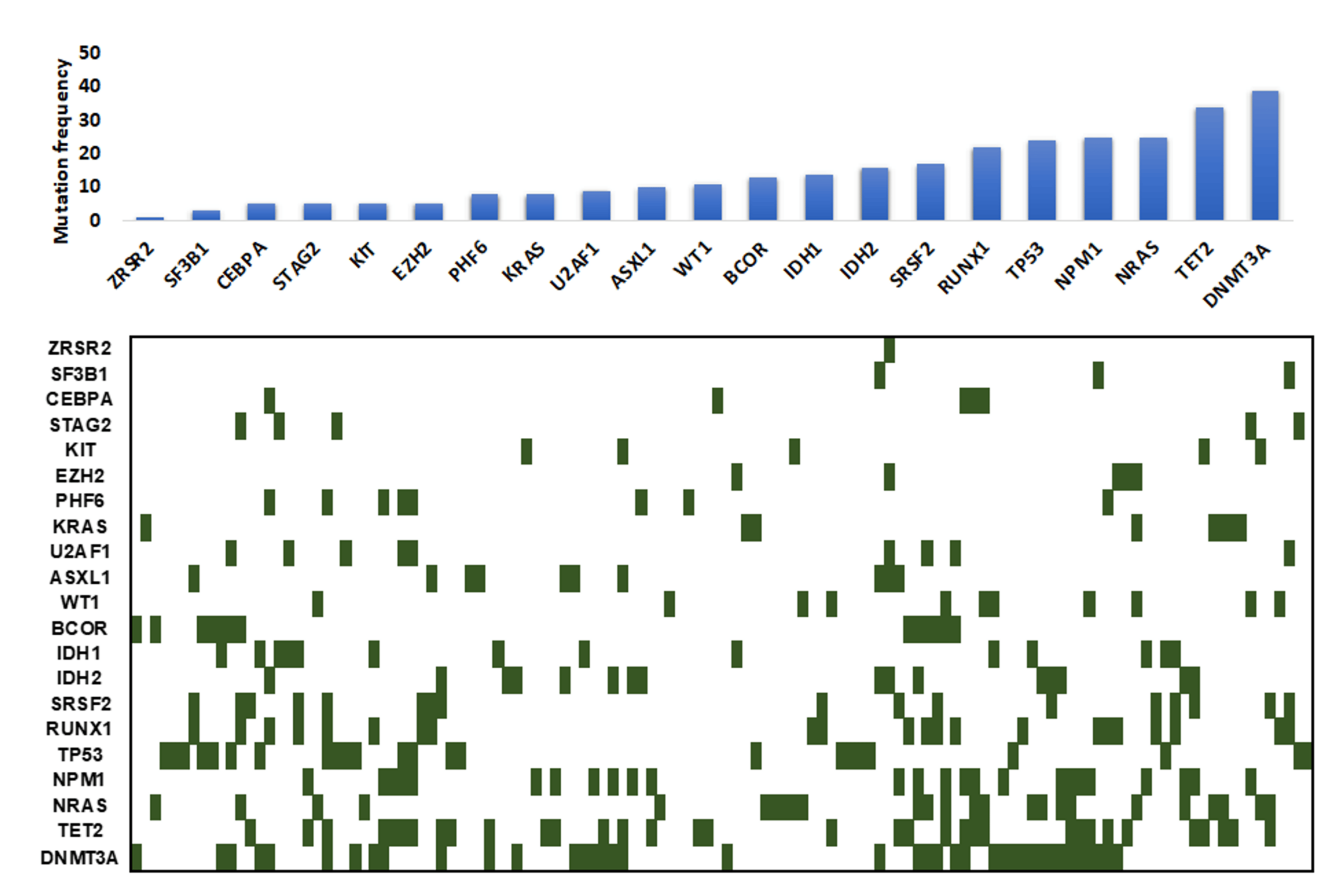
Mutation distribution in 163 Hispanic acute myeloid leukemia patients from Puerto Rico

A total of 163 Hispanic AML patients tested with either AML molecular profile or myeloid molecular profile were identified in our database. Median age was 63 years (range 50-76). Twenty-six (15.95%) patients showed no detectable mutations and 137 (84.05%) showed at least one mutation in the following 21 genes by NGS. The frequencies of these mutations are listed as the following (Table *2* and Figure *1*): *DNMT3A* (23.93%), *TET2* (20.86%), *NRAS* (15.34%), *NPM1* (15.34%), *TP53* (14.72%), *RUNX1* (13.50%), *SRSF2* (10.43%), *IDH2* (9.82%), *IDH1* (8.59%), *BCOR* (7.89%), *WT1* (6.75%), *ASXL1* (6.13%), *U2AF1* (5.52%), *KRAS* (4.91%), *PHF6* (4.91%), *EZH2* (3.07%), *KIT* (3.07%), *STAG2* (3.07%), *CEBPA* (3.07%), *SFR3B1* (1.84%), *ZRSR2* (0.61%). Figure *1* summarizes mutation distribution in 137 positive patients.

**Table 2:** Mutation rates in 163 Hispanic acute myeloid leukemia patients from Puerto Rico

Gene	Number of patients with mutation	Frequency
DNMT3A	39	23.93%
TET2	34	20.86%
NRSA	25	15.34%
NPM1	25	15.34
TP53	24	14.72%
RUNX1	22	13.50%
SRSF2	17	10.43%
IDH2	16	9.82%
IDH1	14	8.59%
BCOR	13	7.89%
WT1	11	6.75%
ASXL1	10	6.13%
U2AF1	9	5.52%
KRAS	8	4.91%
PHF6	8	4.91%
EZH2	5	3.07%
KIT	5	3.07%
STAG2	5	3.07%
CEBPA	5	3.07%
SFR3B1	3	1.84%
ZRSR2	1	0.61%

In addition, 338 AML patients were tested for ITD and TKD in *FLT3*. Sixty patients had ITD (17.75%), 18 patients had TKD (5.36%), and 6 patients had both ITD and TKD (1.79%). In MLL, we tested 76 AML patients for *MLL-PTD*. 13 patients had *MLL-PTD* (17.11%) (Table *3*).

**Table 3:** Mutations in FLT3 and MLL

Mutation	Mutational frequency (%)
FLT3-ITD	60/338 (17.75%)
FLT3-TKD	18/338 (5.36%)
MLL-PTD	13/76 (17.11%)

There are two recent publications about molecular mutation profiles in the Hispanic AML population. We compare the mutation frequencies of the Hispanic AML population from PR in our current study with those published by Bradley et al. [6] and Darbinyan et al. [2] (Tables 4-5). Most of the 21 genes analyzed in our cohort show no significant difference compared with the Bradley cohort and Darbinyan cohort. However, the Hispanic AML population from PR has significantly higher mutation rates in *WT1* and *KRAS* genes than the Bradley cohort and a significantly lower mutation rate in the *ASXL1* gene than the Darbinyan cohort.

**Table 4:** Comparison of mutation frequencies in our cohort and in Bradley cohort *p*<0.05: significance; ----: not performed.

Gene	Current cohort, Positive#/Tested# (%)	Bradley cohort, Positive#/Tested# (%)	*P-*value
DNMT3A	39/163 (23.93%)	11/58 (18.97%)	0.4718
TET2	34/163 (20.86%)	10/58 (17.24%)	0.7021
NRAS	25/163 (15.34%)	2/20 (10.00%)	0.7427
NPM1	25/163 (15.34%)	4/58 (6.90%)	0.1172
TP53	24/163 (14.72%)	3/20 (15.00%)	1
RUNX1	22/163 (13.50%)	4/20 (20.00%)	0.4942
SRSF2	17/163 (10.43%)	4/20 (20.00%)	0.2556
IDH2	16/163 (9.82%)	9/58 (15.52%)	0.237
IDH1	14/163 (8.59%)	1/58 (1.72%)	0.237
BCOR	13/163 (7.89%)	2/20 (10.00%)	0.6707
WT1	11/163 (6.75%)	5/20 (25.00%)	0.0186
ASXL1	10/163 (6.13%)	3/58 (5.17%)	1
U2AF1	9/163 (5.52%)	----	----
KRAS	8/163 (4.91%)	4/20 (20.00%)	0.0292
PHF6	8/163 (4.91%)	4/58 (6.90%)	0.5186
EZH2	5/163 (3.07%)	----	----
KIT	5/163 (3.07%)	3/58 (5.17%)	0.4353
STAG2	5/163 (3.07%)	----	----
CEBPA	5/163 (3.07%)	5/53 (9.43%)	0.0681
SF3B1	3/163 (1.84%)	1/20 (5.00%)	0.3731
ZRSR2	1/163 (0.61%)	----	----
FLT3-ITD	60/338 (17.75%)	4/53 (7.55%)	0.0719
FLT3-TKD	18/338 (5.36%)	1/58 (1.72%)	0.3318
MLL-PTD	13/76 (17.11%)	3/53 (5.66%)	0.061

**Table 5:** Comparison of mutation frequencies in our cohort and in Darbinyan cohort *p*<0.05: significance; ----: not performed.

Gene	Our cohort, Positive#/Tested# (%)	Darbinyan cohort, Positive#/Tested# (%)	*P-*value
DNMT3A	39/163 (23.93%)	11/46 (23.91%)	1
TET2	34/163 (20.86%)	11/46 (23.91%)	0.6862
NRAS	25/163 (15.34%)	7/46 (15.22%)	1
NPM1	25/163 (15.34%)	8/46 (17.39%)	0.819
TP53	24/163 (14.72%)	7/46 (15.22%)	1
RUNX1	22/163 (13.50%)	6/46 (13.04%)	1
SRSF2	17/163 (10.43%)	----	----
IDH2	16/163 (9.82%)	7/46 (15.22%)	0.2953
IDH1	14/163 (8.59%)	5/46 (10.87%)	0.5746
BCOR	13/163 (7.89%)	----	----
WT1	11/163 (6.75%)	----	----
ASXL1	10/163 (6.13%)	9/46 (19.57%)	0.0161
U2AF1	9/163 (5.52%)	----	----
KRAS	8/163 (4.91%)	----	----
PHF6	8/163 (4.91%)	----	----
EZH2	5/163 (3.07%)	----	----
KIT	5/163 (3.07%)	----	----
STAG2	5/163 (3.07%)	----	----
CEBPA	5/163 (3.07%)	1/46 (2.17%)	1
SFR3B1	3/163 (1.84%)	----	----
ZRSR2	1/163 (0.61%)	----	----
FLT3-ITD	60/338 (17.75%)	6/46 (13.04%)	0.5346
FLT3-TKD	18/338 (5.36%)	----	----
MLL-PTD	13/76 (17.11%)	----	----

## Discussion

Hispanic patients have been reported to have an increased incidence of AML and possibly inferior outcomes compared to non-Hispanics. The disparity seen in the treatment and clinical outcomes of AML in minority groups has been attributed to its clinical and biological heterogeneity. In recent years, a number of articles have been published that have identified common recurrent cytogenetic abnormalities and a number of molecular mutations that have occurred with significantly greater frequency within the Hispanic population than the non-Hispanic population. However, these articles generally had a small sample size (n<100) and were centered in the mainland United States. In this study, we sought to evaluate more data from a primarily Hispanic population center where we believe the data would be more reflective of a Hispanic ethnic group. We have identified the most common recurrent cytogenetic abnormalities and molecular mutations associated with AML prognosis in the Puerto Rican Hispanic population. Identifying the prevalence of these alterations will help physicians advise patients in the fastest-growing ethnic group in the U.S. regarding their treatment options.

Our current study indicates that t(15;17) is the most common recurrent cytogenetic abnormality, followed by inv(16), t(8;21), and t(9;22) in Hispanic AML patients in PR, which are similar to those in the Hispanic population and non-Hispanic white population from the mainland US [2,6,7].

The Hispanic population is not a monolithic racial group, so the genetic admixture within the Hispanic population varies greatly depending on different ancestral regions. We utilized the Fischer exact test to extrapolate the molecular profile of 21 genetic markers from our data and statistically significant differences in mutational frequency among the Hispanic population in different regions. The mutational frequency of *ASXL1* in Puerto Rican Hispanic AML patients was significantly lower than that in the Darbinyan cohort [2], and the mutational frequencies of *WT1* and *KRAS* were significantly lower than that in the Bradley cohort [6]. Hispanic AML populations from different regions likely have different molecular profiles. Hispanic AML populations at Montefiore Medical Center in New York and at Miami Medical Center and Tampa Cancer Institute in Florida may have more diversified genetic backgrounds because they emigrated from different countries, and the Hispanic AML population in PR is relatively homogenous.

*ASXL1* is associated with an adverse prognostic significance in AML patients. The prevalence of *ASXL1* among AML patients was previously found to be 5-30% [8-10]. The differences in the prevalence of *ASXL1* among Hispanics compared to non-Hispanics are disputed, as previous studies have yielded contradictory results. According to Darbinyan et al. [2], *ASXL1* was found to be significantly increased in prevalence among Hispanics in comparison to whites in the Bronx and TCGA cohorts. Our data showed an insignificant increase among Puerto Rican Hispanic AML compared to non-Hispanic white AML in the Bronx and TCGA cohorts (6.13% vs. 3.5%; p=0.3179).

The mutational frequency of *WT1* has been identified in 3.2% to 12.6% of patients with AML [11]. According to Bradley et al. [6], *WT1* was associated with a poor prognosis, which was noted to have a significant increase in prevalence among Hispanics in comparison to non-Hispanic whites. The finding contradicted Monge et al. [3], which noted no difference between the Hispanic and non-Hispanic white populations. Similarly, we did not find a meaningful difference in the mutational frequency of *WT1* in Hispanic and non-Hispanic AML patients in the Bradley cohort [6] (6.75% vs. 6.4%; p=1).

The prevalence of *KRAS* mutations ranges from 2.9% to 5.05% [12-15]. A Chinese study [14] found that *RAS* mutations had no prognostic impact on AML patients. *KRAS* overexpression was a prognostically adverse predictor in AML patients with a normal karyotype [15]. According to Bradley et al. [6], *KRAS* was insignificantly increased in prevalence among Hispanic as opposed to non-Hispanic AML patients. Our study also found that there was no significant increase in prevalence of *KRAS* among Puerto Rican Hispanic as opposed to non-Hispanic AML patients in the Bradley cohort (4.91% vs. 4.3%; p = 1), although Puerto Rican Hispanic AML patients have a significantly higher *KRAS* mutation frequency than Hispanic AML patients in the Bradley cohort.

It is a major limitation since we do not know how the NGS testing results have impacted the treatment and clinical outcomes due to limited access and availability of these patients’ clinical data. Future studies including these clinical data in our cohort will provide more significant findings in this Hispanic AML population.

## Conclusions

Our data shed more light on current knowledge of recurrent cytogenetic and molecular abnormalities in adult Hispanic AML patients. To the best of our knowledge, this is the largest study to date about this abnormality in the adult Hispanic AML population and the first report regarding the frequency of these alterations in adult Puerto Rican Hispanic AML patients. Our current observation reveals no significant difference in mutational frequencies of almost all these genes tested among Hispanic AML patients from different regions in the United States, which is compatible with the previously published studies. The frequencies of common molecular mutations are similar between Puerto Rican Hispanic AML patients and non-Hispanic white AML patients in the mainland USA. Our current data provide a basis for further studies on contributing factor(s) in the disparity of treatment outcomes and clinical outcomes seen among ethnic and racial groups.
